# Public health concerns of highly pathogenic avian influenza H5N1 endemicity in Africa

**DOI:** 10.14202/vetworld.2017.1194-1204

**Published:** 2017-10-08

**Authors:** Olubunmi Gabriel Fasanmi, Ismail Ayoade Odetokun, Fatima Adeola Balogun, Folorunso Oludayo Fasina

**Affiliations:** 1 Department of Production Animal Studies, Faculty of Veterinary Science, University of Pretoria, Onderstepoort, South Africa; 2 Department of Animal Health, Federal Colleges of Animal Health and Production Technology, Ibadan, Nigeria; 3 Department of Veterinary Public Health & Preventive Medicine, University of Ilorin, Ilorin, Nigeria; 4 Emergency Centre for Transboundary Animal Diseases – Food and Agriculture Organisation, Gigiri, Nairobi, Kenya; 5 Department of Veterinary Tropical Diseases, Faculty of Veterinary Science, University of Pretoria, Onderstepoort, South Africa

**Keywords:** Africa, highly pathogenic avian influenza H5N1, public health implications

## Abstract

Highly pathogenic avian influenza virus (HPAIV) H5N1 was first officially reported in Africa in 2006; thereafter this virus has spread rapidly from Nigeria to 11 other African countries. This study was aimed at utilizing data from confirmed laboratory reports to carry out a qualitative evaluation of the factors responsible for HPAI H5N1 persistence in Africa and the public health implications; and to suggest appropriate control measures. Relevant publications were sought from data banks and repositories of FAO, OIE, WHO, and Google scholars. Substantiated data on HPAI H5N1 outbreaks in poultry in Africa and in humans across the world were mined. HPAI H5N1 affects poultry and human populations, with Egypt having highest human cases (346) globally. Nigeria had a reinfection from 2014 to 2015, with outbreaks in Côte d’Ivoire, Ghana, Niger, Nigeria, and Burkina Faso throughout 2016 unabated. The persistence of this virus in Africa is attributed to the survivability of HPAIV, ability to evolve other subtypes through genetic reassortment, poor biosecurity compliance at the live bird markets and poultry farms, husbandry methods and multispecies livestock farming, poultry vaccinations, and continuous shedding of HPAIV, transboundary transmission of HPAIV through poultry trades; and transcontinental migratory birds. There is, therefore, the need for African nations to realistically reassess their status, through regular surveillance and be transparent with HPAI H5N1 outbreak data. Also, it is important to have an understanding of HPAIV migration dynamics which will be helpful in epidemiological modeling, disease prevention, control and eradication measures.

## Introduction

The highly pathogenic avian influenza (HPAI) H5N1 has been described as a highly contagious viral disease in several avian species. The disease is characterized by high morbidity and mortality and could be potentially contracted by humans and other warm-blooded animals thus making it an emerging pandemic of zoonotic importance [1-6]. This is caused by the pathogenic subtypes H5 and H7 but with the circulating H5N1and H7N9 is causing major problems across the globe [7-9]. HPAI virus (HPAIV) H5N1 was first officially reported in Africa in early 2006. Since the first outbreak in Nigeria, this virus has spread rapidly to other African countries. From the first emergence to date, 12 African countries have experienced H5N1 outbreaks in poultry and confirmed human cases have also been reported by Joannis *et al*. [10], Cattoli *et al*. [11], WHO [12], and Food and Agriculture Organization of the United Nations [13]. Studies suggest that there exist an inexplicable relationship between wild birds and domesticated poultry species throughout the various ecological systems where HPAI H5N1 virus has caused outbreaks [14,15]. However, the wild or migratory birds attributed as sole reservoirs for the perpetuation and spreading of HPAI H5N1 in nature may not be absolutely correct after all. Other potential sources suggested include illegal movement of infected poultry and products, multispecies (pig, duck, etc.) farming, and poor biosecurity compliance level in live bird markets (LBMs) [16-21].

HPAI outbreak is known to have negative attendant consequences on the sales of poultry and poultry products and economics of production [22-25]. It has affected directly the per capita protein intake, especially in developing countries leading to an on toward health effect apart from the attendant fatality [12,13].

This review, however, utilized published data from confirmed laboratory reports to carry out a qualitative evaluation of the factors responsible for HPAI H5N1 persistence in Africa and identified probable public health implications on human existence with the aim of suggesting recommendations that can be applied to develop more effective control and preventive strategies.

### HPAI H5N1 Outbreaks in Africa

**Figure-1:**
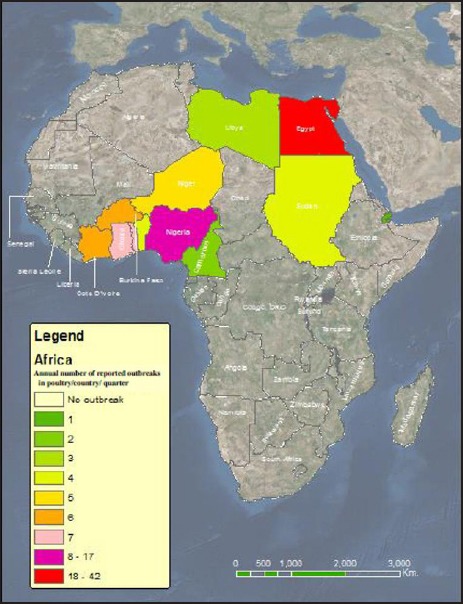
African countries with highly pathogenic avian influenza (HPAI) H5N1 outbreak between 2006 and 2016. Outbreak in this context means sudden occurrence of HPAI H5N1 in poultry farms in a particular locality, state and put together for affected country on a quarterly basis. This is based on outbreak reports only and did not include potential unreported outbreaks. Annual number of reported outbreaks in poultry/country/quarter.

**Figure-2:**
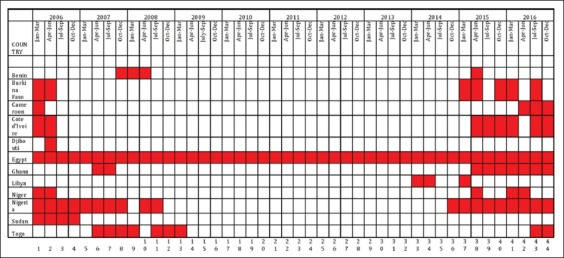
Timeline of highly pathogenic avian influenza (HPAI) H5N1 outbreaks in African countries from 2006 to 2016 http://www.oie.int/animal-health-in-the-world/update-on-avian-influenza/Last update: November 2016.

The HPAI H5N1 was officially reported in Africa, specifically Nigeria in the year 2006, and thereafter other African countries experienced multiple outbreaks, infection, and reinfection affecting millions of birds and a huge economic impact. To date, 12 African countries (Figure-1) have been infected by this deadliest subtype which is believed to be imported from the Asian continent [10,26,27]. The first wave of HPAI H5N1 outbreak started in the year 2006, through 2007and ended in 2008; affecting 8 African countries (Burkina Faso, Cameroon, Cote d’Ivoire, Djibouti, Egypt, Niger, Nigeria, and Sudan) in 2006 and 3 countries (Benin, Ghana, and Togo) in 2007. However, in 2008 no new outbreak was recorded in any other African countries. However, the infection sustained and continued in Benin, and the outbreaks assumed an alarming endemic situation in Egypt. Nigeria and Togo had a reinfection the same year [27,28]. From 26^th^ September 2006, Egypt has been declared as a country endemic to HPAI H5N1, due to the number of confirmed human cases and recorded deaths emanating from these outbreaks [4,29,30]. There was a period of quiescence of HPAI H5N1 outbreak in Africa from 2009 through to 2013, except for the endemicity that persisted in Egypt throughout this period (Figure-2). Another wave of outbreak started in 2014; with Libya recording HPAI outbreak for the first time in history. The subtype of H5N1 isolated from the outbreak was the same lineage as those of the Egyptian counterpart. The political map of Africa shows that Libya shares boundary with the HPAI H5N1 endemic nation of Egypt (Figure-1) which indicates a possibility of transboundary transmission of HPAI H5N1. Toward the last quarter of 2014, Nigeria experienced a reinfection which spanned through 2015 and ushered into Africa a full-blown pandemic of H5N1. This period was characterized by continued outbreaks of HPAI in previously affected countries in Africa. In West Africa, outbreaks and reinfections were however confirmed in Côte d’Ivoire, Ghana, Niger, Nigeria, and Burkina Faso [5,27]. Djibouti and Sudan experienced just single outbreak of HPAI H5N1 between 2006 and 2016, which was reported in 2006; no reinfections were reported in these countries (Figure-2). Considering the spread of H5N1 HPAI in West Africa to three additional countries (Côte d’Ivoire, Ghana, and Niger) during this period of outbreaks, efforts were made at identifying the risk(s) of continued spread within affected countries and spread to non-infected countries in the West African subregion. A conducted survey revealed that the risk of introduction of HPAI H5N1 was estimated as low for Benin and Togo and negligible for Cameroon, Senegal, and Guinea. Burkina Faso, Côte d’Ivoire, and Niger were pinpointed as the most likely routes of viral incursion [28]. These predictions failed to happen as most of the countries mentioned to have negligible to low-risk experienced these outbreaks on a massive scale and are still experiencing it unabated, except Senegal and Guinea that are spared the waves of H5N1 outbreaks and have never experience any outbreak in the past [13]. As at the time of compiling this study, the HPAI H5N1 that started from 2015 is still being experienced actively in the West African subregion (Burkina Faso, Cote d’Ivoire, Ghana, Nigeria, and Togo), Cameroon which is in proximity with Nigeria; and Egypt which is already endemic with HPAI H5N1. Nigeria has been affected by H5N1 HPAI outbreaks in poultry since December 2014 and circulation of the virus is considered to be endemic because it has been non-stop to date [13].

### Factors Responsible for Persistence of HPAI H5N1 in Africa

It has been discovered that the development of large-scale commercial farming will enhance the potential for epidemic transmission and evolution of influenza viruses. High stocking densities in large commercial farms will facilitate rapid and efficient transmission of highly virulent viruses such as H5N1 that might otherwise kill their hosts before being transmitted [31,32]. Researchers at the outset of HPAI outbreaks have shown that repeatedly passing a low pathogenic avian influenza (LPAI) virus among susceptible chickens can lead to the evolution of a highly pathogenic strain [33]. The prolonged and continuous circulation of HPAI H5N1 virus in domestic poultry in Asia and Africa has been alarming. This virus has evolved into multiple genetic lineages that differ antigenically [34]. The Qinghai strain, predominant variant in Africa, has acquired several troubling characteristics such as respiratory rather than fecal transmission in poultry, increased thermal stability, and ability to mutate leading to high pathogenicity in mammals, including humans [7]. Researchers have reiterated several times that there are fewer domestic waterfowls in Africa than in Asia, but it remains unknown whether the available few waterfowls can perpetuate the virus through the warm season; even if they do, that alone does not suffice for the persistence of HPAI H5N1 in Africa. It is also clearly unknown whether HPA1 H5N1 is being maintained in African local birds or periodically reintroduced [35]. Free-ranged flocks have been identified to be more likely exposed to wild birds carrying the LPAI strains rather than commercial poultry flocks, thus providing these free-range birds with constant challenge and immunity maintenance [36]. It has been established that there are other media through which these viruses can be maintained, sustained, and perpetuated in nature, especially in Africa [17-21]. Analyses and comparative assessments of documented data of HPAI H5N1 outbreaks in poultry over a period of 11 years (2006-2016) in Egypt and Nigeria show a progressive increase in the number of outbreaks in poultry; from 2006 to 2008 to the outbreaks in subsequent years [26,28]. This is a reflection and a pointer toward the endemic state of HPAI H5N1, and all these portend a bleak and elusive hope of controlling and eradicating the deadly viral sub-type in the continent of Africa.

### Persistence and perpetuation of HPAIV through evolution or genetic reassortment

Li *et al*. [8] have established that Siberia is the major hub for the dispersal of the influenza viruses, while Southeast Asia and Africa are major sources of genetic and antigenic novel strains, especially HPAI H5N1. They averred further that Africa has the highest persistence and relative genetic diversity; even when extrapolated to 2016. Persistence and, hence, perpetuation of HPAI H5N1 are either reflected as a result of antigenic drift (which tends to cause only small changes in the biological behavior of the virus) or antigenic shift (the exchange of hemagglutinin antigens or neuraminidase antigens) between different influenza A virus subtypes coinfecting a particular host thereby resulting in genetic reassortment; these subtypes have conveniently adapted to both humans and swine, and currently circulate in nature [37-42]. Genetic reassortment can produce new influenza A viruses, such as in the case of the recent pandemic influenza A H5N1, H5N2, and H5N9 outbreaks experienced in France [30,43], H5N1 with different sublineages in Egypt [27,44], and the many HPAI subtypes recorded in Asia after the first outbreak of HPAI in 1996 giving rise to novel virions [45,46]. Furthermore, the emergence of HPAI H5N8 subtype outbreak in 2014 from Eastern China and South Korea [47,48] was confirmed to have evolved from H5N1 [46,49,50]. H5N8 subtype has wrecked unquantifiable havoc in poultry industries worldwide, and has touched 7 countries in Africa (Nigeria, Niger, Cameroon, Togo, Uganda, Zimbabwe, and South Africa (Table-1) [51,52]. Both antigenic drift and genetic reassortment, to varying degrees result in unpredictable changes to virus behavior such as transmissibility, host range, virulence, treatment, and vaccine efficacy [19]. Considering the spate of HPAI H5N1 epidemic, antigenic drift still appears to be the main mechanism, and there is scientific evidence that it has already resulted in changes in virus behavior, thus has affected treatment efficacy in humans [53,54].

**Table-1:** Summary of circulating HPAI subtypes and year of isolation in different African countries.

Country	Subtypes in circulation	Year of isolation and identification of subtype
Algeria	H7N1	2016
Benin	H5N1	2007 and 2015
Burkina Faso	H5N1	2006, 2015 and 2016
Cameroon	H5N1, H5N8	2006, 2016, 2017
Cote d’Ivoire	H5N1	2006, 2015, 2016
Djibouti	H5N1	2006
Egypt	H5N1, H5N8, H9N2*	2006 to 2017 highly endemic with these subtypes
Ghana	H5N1	2007, 2015, 2016
Libya	H5N1	2014, 2015
Niger	H5N1, H5N8	2006, 2015, 2016
Nigeria	H5N1, H5N8	2006, 2007, 2008, 2014 to 2017
South Africa	H5N8	2017
Sudan	H5N1	2006
Togo	H5N1	2007, 2008, 2016
Tunisia	H5N8	2017
Uganda	H5, H5N8	2017
Zimbabwe	H5N8	2017

OIE [51].

* H9N2 may not be classified as HPAI, but it is endemic in Egypt [52].

HPAI=Highly pathogenic avian influenza

### Poor biosecurity compliance at LBMs in Africa

The most important meeting points for all birds kept by people are LBMs. There are different types of LBMs, whose sizes and capacity range from large wholesale to small local markets that operate only occasionally. LBMs also play a key role in the dynamics of influenza virus transmission and evolution. LBMs are suitable vehicles for the rapid dissemination of influenza viruses because of the central role they play and the many trade links they have with farms, roads, abattoirs, households, and many other locations; and they bring together different species of animals from different geographical areas into an unsanitary environment and this can facilitate reassortment between viruses [55]. LBMs have been implicated in a number of avian influenza (AI) outbreaks and in facilitating endemic influenza virus introduction, transmission, and maintenance in circulation worldwide [55-60]. Many of the outbreaks recorded in Nigeria and Egypt are market-based or have links with the LBMs [44,46,61-63]. This was further corroborated by the submissions of Fasanmi *et al*. [21], at the LBMs in Egypt and Nigeria on biosecurity compliance and associated risk factors that could predispose to HPAI H5N1. Five factors (three protective and two risk factors) were compromised and could predispose to HPAI H5N1 outbreak significantly (Table-2) [21]. The display and trade of wild animals and birds in LBMs have been pointed out as another risk factor, because of the possibility of their exposure to low-grade influenza infection or may be reservoirs of infections, and there is possibility of shedding the virus and contaminating the LBMs environment [64,65]. Patterns of spread of this infection have been associated with uncontrolled movement of poultry and poultry products, lack of effective contingency plans to guide the containment, geographical, and ecological factors [28,66].

**Table-2:** Identified risk factors associated with biosecurity compliance level in LBMs, Nigeria, and Egypt. [21]

Variables	Odd ratio	SE	p	95% CI
Wild animals traded in the market	34.90	31.21	0.01	6.05-201.40
Mandatory routine disinfection of LBMs	0.13	0.06	0.00	0.05-0.33
Fencing and gates around the LBMs	0.02	0.03	0.01	0.00-0.32
Hands washing after slaughter	0.41	0.19	0.05	0.17-1.01
Claims of hand disinfection after slaughter	31.16	48.42	0.03	1.48-655.06

LBM=Live bird market, SE=Standard error, CI=Confidence interval

### Poultry husbandry methods and multispecies livestock farming

Poultry production and husbandry methods in developing countries such as Africa is largely dominated by backyard, traditional, or household poultry; representing about 80% of poultry stocks and can be intensive, semi-intensive, or extensive [67-69]. This type of poultry production in African countries often consist of free indigenous breeds, with various species mixed in the same flock [68,70-72]. Poultry closely mingles with humans in the same household as well as with wild birds and other livestock where they are also exposed to vermin. More often than not backyard poultry is characterized by small poultry population with poor biosecurity measures. Biosecurity is considered as an indispensable tool to mitigate the spread of infectious diseases thereby improving the health status [36,73-76]. Poor or lack of disease control strategies and inadequate management practices result in high levels of mortality due to infectious diseases. This could easily have been averted if poultry disease management which involves taking steps to ensure good hygiene. Furthermore, increasing the standards of cleanliness as well as containment when applied reduces or prevent the risk of introducing disease into a flock and hence mortality [67,77-81]. A recent study conducted by Arafa *et al*. [82] and Arafa *et al*. [83] showed that epidemiological dynamics of HPAI has changed with the origins of majority of outbreaks. This points toward household poultry, and it is associated with human cases of H5N1 infections. The rearing of multispecies of poultry (duck, turkey, pigeon, quail, and chicken) and other livestock is done in the same compound with human habitations under very poor biosecurity measures is a common practice in most countries of Africa, especially Nigeria and Egypt. Operating under such poultry production setting creates an avenue for the perpetuation of already existing HPAI viral subtypes. This provides an opportunity for reassortment for the formation of Novel and new subtypes and sublineages of HPAI in Africa. A similar scenario played out recently in Europe, specifically France, from December 2015 to date. A total of 81 outbreaks of HPAI were recorded mainly in extensively reared duck farms, initially harboring LPAI viruses. However, due to very poor biosecurity measures and multispecies rearing, these viruses eventually reassorted and within the spate of few months generated three HPAI subtypes (H5N1, H5N2, and H5N9). These eventually spread to commercial layer farms, killing millions of chickens, and ducks [43,84,85].

### Vaccination and continuous shedding of HPAIV in the environments

Whenever there is an outbreak of HPAI, there are two primary strategies to control and possibly prevent further spread of the infection; these are vaccination and depopulation or stamping out. Depopulation is very expensive to implement, but it is the method of choice when epidemics of highly pathogenic influenza strikes within poultry population in a pen house [86]. Depopulation was practiced one time or the other in 11 out of the 12 African countries that have experienced HPAI H5N1 outbreaks. Egypt combined both depopulation and vaccination. Vaccination, on the other hand, is often undertaken as a preventative measure, although it has also been shown to be effective during outbreaks of H5N1 in the Asian continent and in Middle East [87-90].

The rationale behind the use of HPAI vaccine is that it is the only additional measure that can be taken to attempt to reduce disease spread. The expected results of the implementation of a vaccination policy on the dynamics of infection are primarily those of reducing the susceptibility to infection (using a higher dose of viral antigen to establish productive infection). Furthermore, reduction in viral load shed in the environment and also complying with DIVA (differentiation between infected and vaccinated animals) strategy. Increased biosafety regulations both at vaccine production centers as well as on the field are very necessary, to help in the eradication of HPAI H5N1 [90,91]. Egypt chose to adopt HPAI H5N1 vaccination taking into account the aforementioned reasons. However, 9 years after the preferred choice of AI vaccination was instituted, the impact on disease control of AI vaccination has been very limited. Despite the continuous vaccination of poultry against HPAI, poultry outbreaks and human cases are reported regularly unabated, hence maintained the endemic status [30,89,92] (Figure-2). Even in the face of the present endemic situation in Egypt, Kilany *et al*. [93] used recombinant Turkey Herpes virus and attenuated H5N1vaccines to stimulate the production of high antibody titer which protected Mulard ducks against HPAI H5N1. The current vaccination program and its lack of positive impact on the spread of infection or the maintenance of public health safety may possibly be due to the poor quality of vaccines. Poor techniques and/or incorrect administration of the HPAI H5N1 vaccinations and a mismatch between the antigens in the vaccine and the antigens circulating in the wild are other factors which can lead to a limited impact as a disease control measure [89,94-96]. The direct effect of this failure is at the expense of the human populace because the birds would have assumed the status of a carrier, constantly shedding live viruses in the same environment where humans are accommodated. Live AI vaccines prepared using natural avirulent or attenuated strains are associated with an inherent risk for generating new reassortant influenza viruses. This can lead to the evolution of viruses with an increased virulence with unpredictable characteristics [97,98]. These reassortants could arise when a host bird is simultaneously infected with both live AI vaccine and another influenza virus; that may arise as a result of poor biosecurity measures [89,90,99,100]. Evidence has shown that the majority of the viruses derived from vaccinated poultry farms in Egypt belonged to clade 2.2.1.1 and recently, a novel cluster of clade 2.2.1.2 was identified to have emerged which probably has human health impact [30,76].

### The ability of HPAIV to survive and persist in an environment

Ordinarily, HPAIV can survive in cool and moist conditions, particularly when organic material is present [101]. The World Organization for Animal Health (OIE) states the following about the resistance of AI viruses to physical and chemical actions [5]:

- High temperature over a period of time (pasteurization and cooking) can inactivate the HPAIV in infected poultry meat and egg. Cooking whole eggs at 60°C for over 3 and 9 min for poultry meat will inactivate the virus. Cooking meat for a temperature of 70°C for 1 min will also inactivate the virus. The HPAI virus can survive indefinitely if poultry product is well frozen.
- HPAIV is inactivated by acidic pH, usually <2.
- They are inactivated in the presence of certain chemicals; organic solvents and detergents such as sodium desoxycholate and sodium dodecyl sulfate. When organic matter is present aldehydes, β-propiolactone, and binary ethylenimine should be used for inactivation. After organic matter has been removed, phenolics, quaternary ammonium compounds, and oxidizing agents (such as sodium hypochlorite).
- HPAIV are inactivated by disinfectants, clean surfaces with no organic matter, sodium hypochlorite (5.25%) will be enough, sodium hydroxide (2%), phenols, acidified ionophores, chlorine dioxide, or strong oxidizing agents [40].
- Survivability:
  - Can survive in surface waters 26-30 days at 28°C and 94-158 days at 17°C.
  - Viable in liquid feces for 30-35 days at 4°C and for 7 days at 20°C.
  - Can survive for 4 days in chicken feces held between 25 and 32°C in the shade.
  - Composting will kill virus within poultry carcasses in <10 days.

However, the predominant variant of HPAI H5N1 virus in Africa has undergone transformation and acquired several problematic characteristics; such as increased thermal stability and a PB2 gene mutation associated with pathogenicity in mammals, including human beings [7]. This will make the virus to survive even in the face of harsh and inclement African weather conditions and continue to persist in the environment.

The persistence of HPAI H5N1 in Africa can also be enhanced through transboundary transmission of the pathogenic viruses, especially among the West African countries and some parts of northern Africa. This can occur through informal poultry trades and, live and migratory bird movement [26]. In a recent survey conducted by Food and Agriculture Organization [13], between Cameroonian regions (with HPAI H5N1 outbreaks) and unaffected Central African countries, it was confirmed that illegal trades of poultry, poultry products, and porous borders are factors that predispose to varying levels of risk of transmission of HPAI H5N1. This occurs from infected regions to vulnerable and uninfected neighboring Central African countries, and the likelihood of outbreaks occurring is high.

### HPAI H5N1 Human Infections and Public Health Implications

Following the emergence and series of outbreaks of H5N1 (HPAI) across the globe, this has attracted considerable public and media outcry, because the viruses involved have been shown to be capable of producing fatal disease in humans. This virus is able to change into a highly pathogenic variant with very high fatality rate. Being a very adaptable virus, these spill-over events are frequent, and numerous species are susceptible to influenza virus. When a subtype of AIV that has not previously infected humans crosses the species barrier, adapts to humans, and spreads easily, a pandemic event is imminent. This has given rise to the fear that the virus might acquire the capacity for sustained human-to-human transmission if the viruses gain opportunities to infect and evolve in humans. It may accelerate adaptation of the avian viruses to have more predilections for humans and thus cause a global influenza pandemic. However, to date, no sustained human-to-human transmission has been observed or recorded [1,12,24,92,102]. The evidence and theories which support an influenza A virus to adapt in a mammalian host remain unclear than transition of an influenza A virus from a low pathogenicity to a highly pathogenic form in poultry [39,103].

The majority of human cases of AI (H5N1) infection have been associated with direct or indirect contact with infected live or dead poultry. HPAIV is usually transmitted through direct exposure to HPAI infected birds, feces, or secretions from infected birds. Transmission of the virus can also result from movement of contaminated fomites including by people, on contaminated clothing, equipment, and vehicles. Airborne transmission is not likely a primary mode of transmission, although it may occur over short distances as an aerosol [9,92,104]. There is no evidence that the disease can be spread to people through properly cooked food. At present, there is no known cure for HPAI and immunization remains a complex and cumbersome endeavor. For now, the strategy employed when there are outbreaks is just damage control [3,4]. Three African countries with confirmed human cases include the following; Djibouti, Nigeria, and Egypt as at 2006 [11]. The cumulative number of confirmed human cases for AI H5N1 across the globe per year from 2006 to 2015 is as shown in Table-3. Human cases and fatalities due to influenza A H5N1 virus continue to increase in Egypt, with cases from this country now accounting for the highest number of human cases reported worldwide. This continuous increase of human cases and deaths has been attributed to increase in virus circulation from backyard poultry and exposure to infected poultry. Whenever AI viruses circulate in poultry, sporadic infections, and human cases can occur in people exposed to infected poultry or contaminated environments. Although Egypt has reported an increased number of animal-to-human infections over the past few years, the influenza AI (H5) viruses do not appear to transmit easily among humans, and no sustained human-to-human transmission has been observed [92]. However, Egypt as at the end of 2015 had the highest number of confirmed human cases (346) across the globe, and with 116 deaths recorded, second to Indonesia (Table-3) [12]. The case fatality rate (CFR) in humans due to HPAI H5N1 infection in Egypt is high when compared with other countries. It is worrisome to observe that since the first outbreak in Egypt in 2006, the cases, death and CFR continue to increase unabated [6,92]. This calls for lasting public health strategies to combat the scourge.

**Table-3:** Cumulative number of confirmed human cases and deaths for avian influenza (H5N1) reported to the WHO, 2003-2015.

Country	2003-2009		2010		2011		2012		2013		2014		2015		Total	
							
	Cases	Death	Cases	Death	Cases	Death	Cases	Death	Cases	Death	Cases	Death	Cases	Death	Cases	Death
Azerbaijan	8	5	0	0	0	0	0	0	0	0	0	0	0	0	8	5
Bangladesh	1	0	0	0	2	0	3	0	1	1	0	0	0	0	7	1
Cambodia	9	7	1	1	8	8	3	3	28	14	9	4	0	0	56	37
Canada	0	0	1	1	0	0	0	0	1	1	0	0	0	0	1	1
China	38	25	2	1	1	1	2	1	2	2	2	0	5	1	52	31
Djibouti	1	0	0	0	0	0	0	0	0	0	0	0	0	0	1	0
Egypt	90	27	29	13	39	15	11	5	4	3	37	14	136	39	346	116
Indonesia	162	134	9	7	12	10	9	9	3	3	2	2	2	2	199	167
Iraq	3	2	0	0	0	0	0	0	0	0	0	0	0	0	3	2
Lao People’s Democratic Republic	2	2	0	0	0	0	0	0	0	0	0	0	0	0	2	2
Myanmar	1	0	0	0	0	0	0	0	0	0	0	0	0	0	1	0
Nigeria	1	1	0	0	0	0	0	0	0	0	0	0	0	0	1	1
Pakistan	3	1	0	0	0	0	0	0	0	0	0	0	0	0	3	1
Thailand	25	17	0	0	0	0	0	0	0	0	0	0	0	0	25	17
Turkey	12	4	0	0	0	0	0	0	0	0	0	0	0	0	12	4
Vietnam	112	57	7	2	0	0	0	0	0	0	0	0	0	0	127	64
Total	468	282	48	24	62	34	32	20	39	25	52	22	143	42	844	449

Total number of cases includes number of deaths; the WHO reports only laboratory cases - WHO/GIP, 2015. http://www.who.int/influenza/human_animal_interface/EN_GIP_201503031cumulativeNumberH5N1cases.pdf

### The Way Forward and Preventive Measures

The current outbreak of AI (H5N1) worldwide, especially in developing countries and the emergence and reemergence of novel subtypes is passing a signal of an impending influenza pandemic waiting to explode. Every African nation with or without HPAI H5N1 outbreak must realistically assess its status (especially its borders, live bird markets, and periurban populations). The conduct of regular active surveillance and being transparent with HPAI H5N1outbreak data is very important.

Africa generally, except Egypt is a resource-limited continent, when it comes to infectious and zoonotic diseases, it needs an epidemiological knowledge-based approach; and need to have the knowledge and understanding of migration dynamics of HPAI H5N1 virus in Africa. All these will be helpful for surveillance, disease prevention, and targeting the control measures and potentially eradicating the virus on this continent [8,92].

The enforcement of a control strategy based on culling of birds that are infected or suspected of being infected, based only on the application of sanitary restrictions on farms, may not be sufficient to avoid the spread of HPAI H5N1, particularly in areas that have high poultry population. This is an exercise in futility which results in mass depopulation and remains a huge financial burden and massive loss to stakeholders and national economies [22,25,28]. Furthermore, risk assessments and identification of risk factors predisposing to HPAI H5N1 outbreaks are necessary for modeling and prediction of time and likelihood of locations of future outbreaks [105,106].

The International agencies and donor nations should do more in the area of assistance to African nations with economic recession and that are struggling to survive; because an uncontrolled epidemics of HPAI H5N1 in any nation or continent remains a global threat.

The time has come to set up appropriate measures and put machineries in motion to combat and possibly subdue influenza pandemics; starting from Africa, and to the rest of the world.

### Control Measures

Controlling the disease in animals is the first step in decreasing risks to humans [4], and this will include among others:

- Proper implementation of a vaccination policy on the dynamics of infection that suits a particular country to reduce the susceptibility to infections and the amount of virus shed into the environment. Furthermore, the quality of vaccines, the techniques, and administration of the vaccine must be considered, not to cause more damage.
- All boarders must be well monitored and controlled to prevent smuggling of chicken/birds of unknown status from other countries or countries with reported outbreaks.
- Avoid the importation of fertile eggs, live birds (day-old chicks), and frozen chicken, or turkeys from countries with history/record of HPAI outbreak.
- Proper monitoring of waterways and water bodies to prevent migratory birds (HPAIV carriers) from moving into other countries.
- Promptness in reporting of outbreaks of HPAI and response of the surveillance team also must be swift.
- Adequate payment of compensations for dead and prematurely culled birds following outbreaks in farms or in-contact farms, so as to encourage prompt reporting whenever there are outbreaks.
- Proper monitoring of movement of birds from one area of the country to the other following outbreaks; regulatory government authorities may take preventive action. For instance, by issuing movement restrictions of birds from some parts of the country to limit the spread of this deadly virus during outbreaks.
- Regular active and passive surveillance must be practiced for early detection of HPAIV before they are full-blown; possibly through integrated disease surveillance and response.
- LBMs being a center and meeting point for farmers, should be well monitored, especially for birds entering and exiting market, Biosecurity compliance level must be monitored and enforced.
- Human beings handling poultry or birds should cultivate the habit of “cleanliness is next to godliness” through improved hygiene and wearing of protective apparels.
- Reduction of contacts between domestic birds, wild birds, and human beings; this is key to preventing HPAI infections in human domains.
- Consideration should be made of poultry husbandry methods, especially intensively practiced household poultry in the same location with human habitations. Poultry should be located far from human habitations.
- There is the need to consider, review and assess existing treatment strategies, by adopting symptomatic and definitive treatment methods. Prompt intervention to save human lives whenever bird flu is diagnosed by administering antiviral (for instance Tamiflu^®^), anti-inflammatory agents and immunotherapeutic agents appropriately.

## Conclusion

The HPAI H5N1 virus dwells in Africa, especially the Western and parts of Northern Africa. Other parts of Africa that have not recorded H5N1 outbreaks, recently (2016 and 2017) experienced the outbreak of the novel reassortant subtype (H5N8). There is the need to regulate international trades, monitor borders, adopt, and practice good biosecurity measures in the farms, LBMs, and at homes. Active and passive surveillance must not be compromised, and also all the aforementioned preventive and control measures should be applied before and during HPAI H5N1 outbreaks.

## Authors’ Contributions

OGF: Coordinated the study and drafted the manuscript. FAB and OGF: Searched for relevant literatures. FOF and IAO: Critically reviewed the manuscript. All author read and approved the final manuscript.

## References

[1] Lekcharoensuk P (2008). Highly pathogenic avian influenza (HPAI) H5N1 virus in Asia:Evolution and vaccination. Vet. World.

[2] Kabir S.M.L (2010). Avian flu (H5N1):Threat of 'global pandemic'is growing and it's impact on the developing countries'economy. Afr. J. Microbiol. Res.

[3] Wahlgren J (2011). Influenza a viruses:An ecology review. Inf. Eco. Epidemiol.

[4] WHO, World Health Organization (2014). Avian Influenza Facts Sheets.

[5] OIE, World Organisation for Animal Health (2014). Highly Pathogenic Avian Influenza, Technical Disease Card.

[6] Fasanmi O.G, Laleye A.T, Fasina F.O (2016). Systematic review and meta-analyses of cases and deaths associated with HPAI H5N1 in humans and poultry. CAB Rev. Perspect. Agric. Vet. Sci. Nutr. Nat. Resour.

[7] Peiris J.S.M, de Jong M.D, Guan Y (2007). Avian influenza virus (H5N1):A threat to human health. Clin. Microbiol. Rev.

[8] Li X, Zhang Z, Yu A, Ho S.Y.W, Carr M.J, Zheng W, Zhang Y, Zhu C, Lei F, Shi W (2014). Global and local persistence of influenza A(H5N1) virus. Emerg. Infect. Dis.

[9] USDA, United States Department of Agriculture (2015). Highly Pathogenic Avian Influenza Standard Operating Procedures:Foreign Animal Disease, Preparedness and Response Plan.

[10] Joannis T, Lombin L.H, De Benedictis P, Cattoli G, Capua I (2006). Confirmation of H5N1 avian influenza in Africa. Vet. Rec.

[11] Cattoli G, Monne I, Fusaro A, Joannis T.M, Lombin L.H, Aly M.M, Domenech J.M, Capua I (2009). Highly pathogenic avian influenza virus subtype H5N1 in Africa:A comprehensive phylogenetic analysis and molecular characterization of isolates. PLoS One.

[12] WHO, World Health Organization (2015). Cumulative Number of Confirmed Human Cases for Avian Influenza A(H5N1) Reported to WHO, 2003-2015.

[13] Food and Agriculture Organization of the United Nations. Worries Rise Over Outbreaks of Avian Flu in West Africa. News Article.

[14] Sims L.D, Domenech J, Benigno C, Kahn S, Kamata A, Lubroth J, Martin V, Roeder P (2005). Origin and evolution of highly pathogenic H5N1 avian influenza in Asia. Vet. Rec.

[15] Alexander D.J, Brown I.H (2009). History of highly pathogenic avian influenza. Rev. Sci. Tech. Int. Off. Epizoot.

[16] Peiris J.S.M, Guan Y, Markwell D, Ghose P, Webster R.G, Shortridge K.F (2001). Co-circulation of Avian H9N2 and contemporary “human” H3N2 influenza a viruses in pigs in southeastern China:Potential for genetic reassortment?. J. Virol.

[17] Gilbert M, Chaitaweesub P, Parakamawongsa T, Premashthira S, Tiensin T, Kalpravidh W, Wagner H, Slingenbergh J (2006). Free-grazing ducks and highly pathogenic avian influenza, Thailand. Emerg. Infect. Dis.

[18] Gauthier-Clerc M, Lebarbenchon C, Thomas F (2007). Recent expansion of highly pathogenic avian influenza H5N1:A critical review. Proc. Natl. Acad. Sci. United States Am.

[19] Pfeiffer D.U, Otte M.J, Roland-Holst D, Inui K, Tung N, Ziberman D (2011). Implications of global and regional patterns of highly pathogenic avian influenza virus H5N1 clades for risk management. Vet. J.

[20] Kuchipudi S.V, Tellabati M, Sebastian S, Londt B.Z, Jansen C, Vervelde L, Brookes S.M, Brown I.H (2014). Highly pathogenic avian influenza virus infection in chickens but not ducks is associated with elevated host immune and pro-inflammatory responses. Vet. Res.

[21] Fasanmi O.G, Ahmed S.S.U, Oladele-Bukola M.O, El-Tahawy A.S, Elbestawy A.R, Fasina F.O (2016). An evaluation of biosecurity compliance levels and assessment of associated risk factors for highly pathogenic avian influenza H5N1 infection of live-bird-markets, Nigeria and Egypt. Acta. Trop.

[22] Rushton J, Viscarra R, Guerne-Bleich E, McLeod A (2005). Impact of avian influenza outbreaks in the poultry sectors of five south East Asian countries (Cambodia, Indonesia, Lao PDR, Thailand and Vietnam):Outbreak costs, responses and potential long-term control. Worlds Poult. Sci. J.

[23] Nicita A (2007). Avian influenza and poultry trade. Policy Research Working Paper 4551.

[24] Otte J, Hinrichs J, Rushton J, Roland-Holst D, Zilberman D (2008). Impacts of avian influenza virus on animal production in developing countries. CAB Rev PAVSNNR.

[25] Fasina F.O, Sirdar M.M, Bisschop S.P.R (2008). The financial cost implications of the highly pathogenic notifiable avian influenza H5N1 in Nigeria. Onderstepoort J. Vet. Res.

[26] FAO, Food and Agriculture Organization of the United Nations (2015). H5N1 HPAI spread in Nigeria and increased risk for neighbouring countries in West Africa. EMPRES Watch.

[27] OIE, World Organisation for Animal Health (2015). Update on Highly Pathogenic Avian Influenza in Animals (Type H5 and H7).

[28] FAO, Food and Agriculture Organization of the United Nations (2015). Global Animal Disease Intelligence Report. Rome, Italy.

[29] Kayali G, Webby R.J, Xiong X, Sherif L.S, El-Ghafar E.A, Ali M.A (2010). Prospective study of avian influenza transmission to humans in Egypt. BMC Publ. Health.

[30] OIE, World Organisation for Animal Health (2016). Update on Highly Pathogenic Avian Influenza in Animals (Type H5 and H7).

[31] Perkins L.E.L, Swayne D.E (2001). Pathobiology of a/chicken/Hong Kong/220/97 (H5N1) avian influenza virus in seven *Gallinaceous* species. Vet. Pathol.

[32] MacDonald J.M (2008). The economic organization of U.S. broiler production. Econ. Inf. Bull.

[33] Ito T, Goto H, Yamamoto E, Tanaka H, Takeuchi M, Kuwayama M, Kawaoka Y, Otsuki K (2001). Generation of a highly pathogenic avian influenza a virus from an avirulent field isolate by passaging in chickens. J. Virol.

[34] Webster R.G, Govorkova E.A (2006). H5N1 influenza-continuing evolution and spread. N. Engl. J. Med.

[35] Fasina F.O, Bisschop S.P, Webster R.G (2007). Avian influenza H5N1 in Africa:An epidemiological twist. Lancet Inf. Dis.

[36] Alhaji N.B, Odetokun I.A (2011). Assessment of biosecurity measures against highly pathogenic avian influenza risks in small-scale commercial farms and free-range poultry flocks in the Northcentral Nigeria. Transbound. Emerg. Dis.

[37] Holmes E.C (2010). The comparative genomics of viral emergence. Proc. Natl. Acad. Sci. United States Am.

[38] Taubenberger J.K, Kash J.C (2010). Influenza virus evolution, host adaptation and pandemic formation. Cell. Host. Microb.

[39] Perdue M.L, Swayne D.E (2008). Molecular determinants of pathogenicity for avian influenza viruses. Avian Influenza.

[40] Suarez D.L, Swayne D.E (2008). Influenza a virus. Avian Influenza.

[41] McHardy A.C, Adams B (2009). The role of genomics in tracking the evolution of influenza a virus. PLoS Pathog.

[42] CFSPH, Iowa State University and Institute for International Cooperation in Animal Biologics (2014). High Pathogenicity Avian Influenza.

[43] Stegemen A (2016). France gets to grip with avian influenza. World Poult.

[44] Arafa A, Suarez D.L, Hassan M.K, Aly MM (2010). Phylogenetic analysis of hemagglutinin and neuraminidase genes of highly pathogenic avian influenza H5N1 Egyptian strains isolated from 2006 to 2008 indicates heterogeneity with multiple distinct sublineages. Avian Dis.

[45] Le T.H, Nguyen N.T.B (2014). Evolutionary dynamics of highly pathogenic avian influenza A/H5N1 HA clades and vaccine implementation in Vietnam. Clin. Exp. Vac. Res.

[46] de Vries E, Guo H, Dai M, Rottier P.J.M, van Kuppeveld F.J.M, de Haan C.A.M (2015). Rapid emergence of highly pathogenic avian influenza subtypes from a subtype H5N1 haemagglutinin variant. Emerg. Inf. Dis.

[47] Fan S, Zhou L, Wu D, Gao X, Pei E, Wang T, Gao Y, Xia X (2014). A novel highly pathogenic H5N8 avian influenza isolated from from wild duck in China. Influenza Other Respir. Viruses.

[48] Jeong J, Kang H.M, Lee E.K, Sung B.M, Kwon Y.K, Kim H.R, Choi K.S, Kim J.Y, Lee H.J, Moon O.K (2014). Highly pathogenic avian influenza virus (H5N8) in domestic poultry and its relationship with migratory birds in South Korea during 2014. Vet. Microbiol.

[49] Wu H, Peng X, Xu L, Jin C, Cheng L, Lu X (2014). Novel reassortant influenza A(H5N8) viruses in domestic ducks, eastern China. Emerg. Infect. Dis.

[50] Lee Y.J, Kang H.M, Lee E.K, Song B.M, Jeong J, Kwon Y.K (2014). Novel reassortant influenza a(H5N8) viruses, South Korea. Emerg. Infect. Dis.

[51] OIE, World Organisation for Animal Health (2017). Update on Avian Influenza in Animals (Types H5 and H7).

[52] Kayali G, Kandeil A, El-Shesheny R, Kayed A.S, Maatouq A.M, Cai Z, McKenzie P.P, Webby R.J (2016). Avian influenza A(H5N1) virus in Egypt. Emerg. Infect. Dis.

[53] Le M.T, Wertheim H.F, Nguyen H.D, Taylor W, Hoang P.V, Vuong C.D, Nguyen H.L, Nguyen H.L, Nguyen H.H, Nguyen T.Q, Nguyen T.V, Van T.D, Ngoc B.T, Bui T.N (2008). Influenza A H5N1 clade 2.3.4 virus with a different antiviral susceptibility profile replaced clade 1 virus in humans in northern Vietnam. PLoS One.

[54] Boltz D, Douangngeun B, Phommachanh P, Sinthasak S, Mondry R, Obert C, Seiler P, Keating R, Suzuki Y, Hiramatsu H, Govorkova E.A, Webster R.G (2010). Emergence of H5N1 avian influenza viruses with reduced sensitivity to neuraminidase inhibitors and novel reassortants in Lao people's democratic republic. J. Gen. Virol.

[55] FAO, Food and Agriculture Organization of the United Nations (2008). Global Programme for the Prevention and Control of H5N1 Highly Pathogenic Avian Influenza.

[56] Kung N.Y, Guan Y, Perkins N.R, Bissett L, Ellis T, Sims L, Morris R.S, Shortridge K.F, Peiris J.S (2003). The impact of a monthly rest day on avian influenza virus isolation rates in retail live poultry markets in HongKong. Avian Dis.

[57] Webster R.G (2004). Wet markets-a continuing source of severe acute respiratory syndrome andinfluenza?. Lancet.

[58] Wang M, Di B, Zhou D.H, Zheng B.J, Jing H, Lin Y.P, Wu X.W, Qin P.Z, Wang Y.L, Jian L.Y (2006). Food markets with live birds as source of avian influenza. Emerg. Infect. Dis.

[59] Indriani R, Samaan G, Gultom A, Loth L, Irianti S, Indryani S, Adjid R (2010). Environmental sampling for avian influenza virus A (H5N1) in live-bird markets, Indonesia. Emerg. Infect. Dis.

[60] Wan X, Dong L, Lan Y, Long L, Xu C, Zou S, Wen L, Cai Z, Wang W, Li X (2011). Indications that live poultry markets are a major source of human infection in China. J. Virol.

[61] Pagani P, Abimiku Y, Emeka-Okolie W (2008). Assessment of the Nigerian Poultry Market Chain to Improve Biosecurity.

[62] Fasina F.O, Njage P.M.K, Ali A.M.M, Yilma J.M, Bwala D.G, Rivas A.L, Stegeman A.J (2015). Development of disease specific, context specific surveilance models:Avian influenza (H5N1)-related risks and behaviours in African countries. ZPH.

[63] ElMasry I, El-Sheikh H, Abdelnabi A, Saad A, Arafa A, Fasina F.O, Lubroth J, Jobre YM (2017). Avian influenza H5N1 surveillance and its dynamics in poultry in live bird markets. Egypt Transbound Emerg. Dis.

[64] Cardona C, Yee K, Carpenter T (2009). Are live bird markets reservoirs for avian influenza?. Poult. Sci.

[65] CDC, Centre for Disease Control and Prevention (2015). Avian Influenza in Birds. Avian Influenza in Wild Birds CDC 24/7.

[66] Rivas A.L, Chowell G, Schwager S, Fasina F.O, Hoogesteijn A.L, Smith S.D, Bisschop S.P.R, Anderson K.L (2010). Lessons from Nigeria:The role of roads in the geo-temporal progression of avian influenza (H5N1) virus. Epidemiol. Infect.

[67] Sonaiya E.B, Swan S.E.J (2004). Small-Scale Poultry Production.

[68] Pym R, Guerne B.E, Hoffmann I (2006). The relative contribution of indigenous chicken breeds to poultry meat and egg production and consumption in the developing countries of Africa and Asia. 12^th^ European Poultry Conference.

[69] Sonaiya F, Smallholder family poultry as a tool to initiate rural development (2007). International Conference Poultry in the Twenty-First Century:Avian Influenza and Beyond.

[70] Minga U, Msoffe P.L, Gwakisa P.S Biodiversity (variation) in disease resistance and in pathogens within rural chicken population. In:22^nd^World's Poultry Congress:June 8-12.

[71] Singh D.P, Fotsa J.C (2011). Opportunities of poultry breeding programmes for family production in developing countries:The bird for the poor. In:E-Conference of the International Network for Family Poultry Development, 24 January, 18 February.

[72] Gueye E.F (2005). Gender aspects in family poultry management systems in developing countries. Worlds Poult. Sci. J.

[73] Dorea F.C, Berghaus R, Hofacre C, Cole D.J (2010). Surveys of biosecurity protocols and practices adopted by growers on commercial poultry farms in Georgia, USA. Avian Dis.

[74] Julien D, Thompson S (2011). Interactive methods to educate and engage poultry producers on the importance of practicing on-farm Biosecurity. J. Agric. Ext. Rural Dev.

[75] Zavala L.A (2011). Viral Respiratory Diseases of Poultry: A Continuous Challenge.

[76] Conan A, Goutard F.L, Sorn S, Vong S (2012). Biosecurity measures for backyard poultry in developing countries:A systematic review. BMC Vet. Res.

[77] Abdelqader A, Wollny C.B, Gauly M (2007). Characterization of local chicken production systems and their potential under different levels of management practice in Jordan. Trop. Anim. Health Prod.

[78] Badubi S.S, Ravindran V, Reid J (2004). A survey of small-scale broiler production systems in Botswana. Trop. Anim. Health Prod.

[79] Biswas P.K, Uddin G.M, Barua H, Roy K, Biswas D, Ahad A, Ahed A, Debnath N.C (2008). Survivability and causes of loss of broody-hen chicks on smallholder households in Bangladesh. Prev. Vet. Med.

[80] Bell J.G Factors limiting production efficiency and profitability from smallholder poultry production. Worlds Poult. Sci. J.

[81] Fasina F.O, Ali A.M, Yilma J.M, Thieme O, Ankers P (2012). The cost-benefit of biosecurity measures on infectious diseases in the Egyptian household poultry. Prev. Vet. Med.

[82] Arafa A, Naguib M, Luttermann C, Selim A, Kilany W, Hagag N, Samy A, Abdelhalim A, Hassan M.K, Abdelwhab E.M, Makonnen Y (2015). Emergence of a novel cluster of influenza A (H5N1) virus clade 2.2. 1.2 with putative human health impact in Egypt. Eurosurveillance.

[83] Arafa A, El-Masry I, Khoulosy S, Hassan M.K, Soliman M, Fasanmi O.G, Fasina F.O, Dauphin G, Lubroth J, Jobre Y.M (2016). Predominance and geo-mapping of avian influenza H5N1 in poultry sectors in Egypt. Geospat. Health.

[84] CIDRAP, Centre for Infectious Disease Research and Policy (2016). Avian Flu in Germany and France.

[85] European Commission, EC (2016). Avian Influenza:Latest Development.

[86] Capua I, Alexander D.J (2009). Avian influenza infection in birds:A challenge and opportunity for the poultry veterinarian. Poult. Sci.

[87] Ellis T.M, Leung C, Chow M.K.W, Bissett L.A, Wong W, Guan Y, Peiris J.S.M (2004). Vaccination of chickens against H5N1 avian influenza in the face of an outbreak interrupts virus transmission. Avian Pathol.

[88] Toro H, Tang D.C (2009). Protection of chickens against avian influenza with nonreplicating adenovirus-vectored vaccine1. Poult. Sci.

[89] Peyre M, Samaha H, Makonnen Y.J, Saad A, Abd-Elnabi A, Galal S, Ettel T, Dauphin G, Lubroth J, Roger F, Domenech J (2009). Avian influenza vaccination in Egypt:Limitations of the current strategy. J. Mol. Genet. Med.

[90] Hilleman M.R (2002). Realities and enigmas of human viral influenza:Pathogenesis, epidemiology and control. Vaccine.

[91] Yakubu B, Nok A.J, Owolodun O.A, Luka P.D, Umaru D.A (2015). The re-occurrence of H5N1 outbreaks necessitates the development of safe and effective influenza vaccine technologies for the prevention and control of avian influenza in Sub-Saharan Africa. Biotech. Mol. Biol. Rev.

[92] ECDC, European Centre for Disease Prevention and Control (2015). Human Infection with Avian Influenza A (H5N1) Virus, Egypt-First.

[93] Kilany W.H, Safwat M, Mohammed S.M, Salim A, Fasina F.O, Fasanmi O.G, Shalaby A.G (2016). Protective efficacy of recombinant turkey herpes virus (rHVT-H5) and inactivated H5N1 vaccines in commercial mulard ducks against the highly pathogenic avian influenza (HPAI) H5N1 clade 2.2.1 virus. PLoS One.

[94] FAO, Food and Agriculture Organization of the United Nations (2011). Approaches to Controlling, Preventing and Eliminating H5N1 Highly Pathogenic Avian Influenza in Endemic Countries.

[95] Zhang H, Wang L, Compans R.W, Wang B.Z (2014). Universal influenza vaccines, a dream to be realized soon. Viruses.

[96] Joost D.P (2008). Avian Influenza in Zoo and Wild Animal Medicine.

[97] Savill N.J, Rose S.G, Keeling M.J, Woolhouse M.E (2006). Silent spread ofH5N1in vaccinated poultry. Nature.

[98] Iwami S, Suzuki T, Takeuchi Y Paradox of vaccination:Is vaccination really effective against avian flu epidemics?. PLoS One.

[99] Webster R, Peiris M, Chen H, Guan Y (2006). H5N1 outbreaks and enzootic influenza. Emerg. Infect. Dis.

[100] Chen H, Smith G, Li K, Fan S.H, Rayner J.M, Vijaykrishna D, Cheung C.L, Huang K (2006). Establishment of multiple sublineages of H5N1 influenza virus in Asia:Implications for pandemic control. Proc. Natl. Acad. Sci.

[101] Swayne D.E (2015). Epidemiology of avian influenza in agricultural and other man-made systems. Avian Influenza.

[102] Ungchusak K.P, Auewarakul S.F, Dowell R, Kitphati W, Auwanit P, Puthavathana M, Uiprasertkul M, Boonnak K, Pittayawonganon C, Cox N.J (2005). Probable person-to-person transmission of avian influenza A (H5N1). N. Engl. J. Med.

[103] Kaplan B.S, Webby R.J (2013). The avian and mammalian host range of highly pathogenic avian H5N1 influenza. Virus Res.

[104] United Nations (UN) Food and Agriculture Organization (FAO) (2015). Questions and Answers:The Facts of Bird Flu.

[105] Stevens K.B, Costard S, Métras R, Pfeiffer D.U (2009). Controlling Avian Flu and Protecting People's Livelihoods in Africa and Indonesia Using Multicriteria Decision Modelling HPAI Research Brief No. 7.

[106] Martin V, Pfeiffer D.U, Zhou X, Xiao X, Prosser D.J, Guo F, Epprecht M, Boles S (2011). Spatial distribution and risk factors of highly pathogenic avian influenza (HPAI) H5N1 in China. PLoS Pathog.

